# Autism spectrum disorder in a patient with Nicolaides-Baraitser Syndrome: case report

**DOI:** 10.31744/einstein_journal/2023RC0480

**Published:** 2023-10-26

**Authors:** Derek Chaves Lopes, Lorenna Sena Teixeira Mendes, Inês Catão Henriques Ferreira

**Affiliations:** 1 Escola Superior de Ciências da Saúde Brasília DF Brazil Escola Superior de Ciências da Saúde , Brasília , DF , Brazil .; 2 Child and Adolescent Psychiatry Residency Program Fundação de Ensino e Pesquisa em Ciências da Saúde Brasília DF Brazil Child and Adolescent Psychiatry Residency Program , Fundação de Ensino e Pesquisa em Ciências da Saúde , Brasília , DF , Brazil .

**Keywords:** Autism spectrum disorder, Nicolaides Baraitser syndrome, Neurodevelopmental Disorders, Brazil

## Abstract

Nicolaides-Baraitser Syndrome is a rare genetic condition that clinically presents with intellectual disabilities, facial and bone changes, and sparse hair. In Brazil, only one case has been previously reported without genetic confirmation. We present the case of an 8-year-old boy, clinically and genetically diagnosed with Nicolaides-Baraitser Syndrome, who developed autism spectrum disorder characteristics with a formal diagnosis at the age of eight. Diagnosing autism spectrum disorder in patients with intellectual disabilities is a clinical challenge requiring careful evaluation.

## INTRODUCTION

Nicolaides-Baraitser Syndrome (NBS) was first described in 1993 based on the symptoms of intellectual disability, facial changes, sparse hair, and bone changes. ^( 1 )^ This rare condition is characterized by a *de novo* missense mutation or deletion in the *SMARCA2* gene, and its phenotype usually progresses throughout life. ^( 2 , 3 )^

Behavioral changes in these patients are described as part of the syndrome, although few cases of autism spectrum disorder have been formally defined. ^( 3 , 4 )^ In 2011, Gana et al. reported two unrelated cases of NBS with changes in social, communicative, and behavioral development. ^( 5 )^

Here, we present the case of a child with a clinical and genetic diagnosis of NBS associated with autism spectrum disorder.

## CASE REPORT

The patient was an 8-year-old boy, son of non-consanguineous parents, delivered by an uncomplicated cesarean section at 39 weeks, with a birth weight of 3,130g, head circumference of 32cm (p3), and APGAR scores of 9 and 10 for the 1st and 5th minutes. He was exclusively breastfed until the 5th month when he faced a challenging introduction to complementary food, which was later evaluated as food texture hypersensitivity. Delayed weight and height growth were observed during childhood, with both measures <p3 until 4 years of age. The patient started walking at 1 year and 3 months.

The patient was referred to various pediatric specialties. Clinical evaluations throughout early childhood revealed cryptorchidism, triangular face, down-slanted palpebral fissures, megalocornea, micrognathia, anterior open bite, a generalized diastema, oral motor hypotonia, and delayed tooth eruption. A dermatological examination revealed xeroderma with mild eczema in the antecubital and popliteal fossae and little fat distribution on the face, rendering a wrinkled skin appearance when smiling. Oropharyngeal dysphagia, gastroesophageal reflux disease, cow milk allergy, and disaccharide intolerance were also diagnosed. He underwent echocardiography, total abdominal ultrasonography, audiometry, and cranial magnetic resonance imaging, revealing no abnormalities.

Genetic investigations were initiated by karyotyping and genetic testing for fragile X, yielding negative results. Next, a comparative genomic hybridization array was performed, which showed two microdeletions of unknown clinical significance: 0.073Mb in 4q31.23 and 0.101Mb in 13q12.12. A parental investigation was conducted with maternal chromosomal microarray analysis, which did not identify any changes, and paternal chromosomal microarray analysis, which identified the same microdeletion as the child at 4q31.23. Plasma amino acid chromatography results were negative at 2 years of age. In 2018, whole exome sequencing revealed a heterozygous pathogenic variant in the *SMARCA2* gene (NM_003070.5:C3314G>C; chr9:2110275 [GRCh 37/Hg]) of autosomal dominant inheritance. This finding, associated with clinical signs of facial dysmorphism, sparse hair, long and thin patterns, and neurodevelopmental delay, is consistent with Nicolaides-Baraitser syndrome.

At 2 years and 5 months of age, the patient was diagnosed with autism and intellectual disability. The patient exhibited difficulty in communication and social interaction, with little interest in playing and eating. Among the symptoms, he had *deficits* in functional language and non-verbal communicative behaviors, irritability and crying in environmental transitions, psychomotor agitation, food selectivity, non-functional play with objects ( *e.g* ., sticks and straws), and the presence of stereotyped motor movements ( *e.g* ., flapping and walking on tiptoe). Because of the presence of target symptoms that did not improve with psychosocial approaches, risperidone was initiated.

The psychiatric follow-up of the patient was regularly maintained until 2020, when he was lost to follow-up. The patient returned to psychiatric care at age 8, when the diagnosis was upheld using the Diagnostic and Statistical Manual of Mental Disorders 5th edition criteria and nomenclature, namely, level III autism spectrum disorder with intellectual impairment, language impairment, and associated with a known genetic condition (NBS).

Informed consent was obtained from the patient’s guardian, and the project was approved by the Ethics Committee of the *Fundação de Ensino e Pesquisa em Ciências da Saúde* (FEPECS) under CAAE: 64399522.6.0000.5553; # 5.817.731.

## DISCUSSION

In this report, we present the case of an 8-year-old Brazilian patient with a clinical and molecular diagnosis of NBS. To our knowledge, this is the first Brazilian report of this syndrome confirmed using whole exome sequencing. ^( 6 )^ Previously, a Brazilian case of a patient with a clinical diagnosis of the syndrome but without genetic testing was reported in the literature. ^( 7 )^

Consistent with the review of cases by Sousa et al. and Abdul-Rahman et al., ^( 3 , 4 )^ the main characteristics of this syndrome are intellectual disability, microcephaly, a triangular face, sparse hair, brachydactyly, prominent interphalangeal joints, and behavioral problems.

In addition to the striking clinical characteristics, NBS tends to be genetically similar, as almost all diagnosed have a *de novo* mutation in the *SMARCA2* gene. Performing chromosomal microarray analysis on the parents confirmed the de novo origin of this mutation in accordance with the genetic etiology already described. ^( 2 , 3 )^ The genetic diagnosis is of vital importance, considering the nonspecificity of many clinical features of NBS. The cardinal craniofacial aspects of the syndrome are more subtle in younger people, and delays in psychomotor development are not specific to NBS. ^( 3 )^

Characterization of autism spectrum disorder in patients with intellectual disabilities is a clinical challenge because of the overlap of some characteristics and difficulties using standardized instruments. ^( 6 )^ Moreover, many medical conditions reported by patients with autism, such as gastrointestinal and immunological issues and speech disorders, are also found in patients with NBS. ^( 8 )^ Nevertheless, even if differentiation is important in aligning treatment expectations, ^( 5 , 6 )^ this should not delay treatment, which should focus mainly on managing problem behaviors and strategies to improve communication and autonomy.

Unlike most previous case reports, the diagnosis of autism was formally made by a child psychiatrist, fulfilling the criteria proposed by the Diagnostic and Statistical Manual of Mental Disorders 5th edition. ^( 9 )^ In this case, there were *deficits* in communication and social interaction in various contexts as well as in socioemotional reciprocity.

## CONCLUSION

To our knowledge, this is the first report of a Nicolaides-Baraitser Syndrome case in Brazil confirmed by genetic testing and a thorough psychiatric evaluation confirming an autism diagnosis. In patients with genetic syndromes and intellectual disabilities, the presence of autism spectrum disorder-like symptoms and the necessity to differentiate between the two conditions present a clinical challenge.
